# Interocular biometric parameters comparison measured with swept-source technology

**DOI:** 10.1007/s10792-021-02020-8

**Published:** 2021-08-21

**Authors:** César Albarrán-Diego, Francisco Poyales, Esther López-Artero, Nuria Garzón, María García-Montero

**Affiliations:** 1 Optics, Optometry and Vision Science Department, Faculty of Physics, University of Valencia, Dr Moliner 50, 46100 Burjassot, Spain; 2 Miranza OA Madrid. C/Galileo 104, 28003 Madrid, Spain; 3 Faculty of Optics and Optometry, Complutense University of Madrid, c/Arcos de Jalón, 118, 28037 Madrid, Spain

**Keywords:** Interocular symmetry, Swept-source, Biometry, IOLMaster 700

## Abstract

**Purpose:**

In the event that any ocular parameter involved in the calculation of intraocular lens power could not be properly measured in one eye, it is important to know whether clinically relevant differences between both eyes can be expected. The aim of this work is to evaluate the symmetry of interocular biometric parameters.

**Methods:**

This was a prospective, cross-sectional study involving 4090 subjects. Patients underwent consecutive swept-source optical biometry performed with an IOLMaster 700 (Carl Zeiss Meditec AG, Jena, Germany). The biometric parameters that were evaluated were: axial length (AL), mean anterior curvature (Rm), anterior chamber depth (ACD), crystalline lens thickness (LT), central corneal thickness (CCT) and white-to-white (WTW). The Chang–Waring chord distance (CWC-D) and the Chang–Waring chord angle (CWC-A) were also evaluated.

**Results:**

There is an excellent correlation between both eyes for almost all the biometric parameters under study, with the exception of the CWC. Agreement for AL was better for eyes shorter than 24 mm. The linearity of the OD-vs-OS relationship can be correctly assumed for all parameters (Cusum test: *p* > 0.05 in all cases).

**Conclusion:**

There are no clinically significant interocular differences for the biometric parameters under study, although for all of them, except the LT, statistically significant differences did arise. In the case of AL, moderate differences can be expected in eyes larger than 24 mm.

## Introduction

Numerous ocular symmetry assessment studies have been carried out, concluding that two eyes follow similar patterns regarding several ocular parameters [1–5]. Therefore, we can say that structural symmetry is inherent to both eyes [2]. This ocular symmetry can aid the diagnosis of pathologies, but it could also help to improve postoperative outcomes, among other scenarios. For example, in LASIK refractive surgery or cataract surgery, several studies have concluded that postoperative refraction in the first eye can help predict refraction outcomes in the second eye [5–8].

Ocular biometry is an essential part of preoperative assessment when evaluating a patient undergoing lens replacement surgery. The latest-generation formulas have included an increasing number of parameters—such as white-to-white (W–W) or corneal thickness—with the aim of fine-tuning postoperative outcomes, and the different eye biometers have evolved to newer swept-source optical coherence tomography (OCT) technology [9].

In this context, the goal of this study was to evaluate the symmetry of interocular biometric parameters in a large population, using a biometer endowed with swept-source technology.

## Materials and methods

A total of 4090 patients—2546 female and 1544 male—were included in this study, recruited from the patients undergoing cataract surgery in the center and who met the inclusion criteria.

These patients underwent consecutive swept-source optical biometry performed with an IOLMaster 700 (Carl Zeiss Meditec AG, Jena, Germany). The study followed the tenets of the Declaration of Helsinki and had been approved by the local ethics committee. The assessments took place between February 2014 and December 2018 at Miranza IOA Madrid. The inclusion criteria limited the eligible subject population to phakic patients with no history of ocular surgery or eye trauma. Contact lens wearers had to stop using them at least 15 days before the assessment and after discarding corneal warpage with a new topography.

All measurements were taken before instilling mydriatic eye drops and before measuring intraocular pressure with contact tonometry, to avoid any ocular surface irregularities that could arise secondary to these procedures. Three measurements per eye are obtained by the same operator for each subject when a biometry is performed. The measurements were taken by two of the authors (ELA and NG). The biometric parameters that were evaluated were those that have been included in various IOL power calculation formulas, including: axial length (AL), mean anterior curvature (Rm), anterior chamber depth (ACD), crystalline lens thickness (LT), central corneal thickness (CCT) and white-to-white (WTW). Moreover, the Chang*–*Waring chord distance (CWC-D) and the Chang*–*Waring chord angle (CWC-A) were also evaluated. The CWC-D or chord length μ is the displacement (distance) between the subject-fixated coaxially sighted corneal light reflex and the pupil center, while CWC-A is the orientation (angle) of that displacement (e.g., Chord μ: 0.35 mm @ 55°) [10]. The quality of each measurement was assessed before performing the data analysis, and those eyes that had raised “warning” or “failed” alerts for any of the biometric parameters under assessment were completely excluded from the analysis.

### Statistical analysis

A descriptive and comparative analysis between both eyes was carried out focusing on the above-mentioned biometric parameters. The comparative analysis was carried out by means of either the Student’s *t-*test for paired samples or the Wilcoxon test depending on whether or not the variables met the criteria for normality (assessed by the Shapiro–Wilk test). Furthermore, the intraclass correlation coefficient (ICC) was also analyzed. Aside from the numerical analysis, Bland–Altman graphs were also plotted for each parameter. Moreover, concordance between both eyes was studied analytically using the Passing–Bablok regression to ascertain whether or not left- and right-eye measurements are interchangeable.

Given the vector nature of the CWC—involving a magnitude (CWC-D) and an orientation (CWC-A)—this variable was decomposed into its vertical [*y* = CWC-D*sine(CWC-A)] and horizontal [*x* = CWC-D*cosine(CWC-A)] components to facilitate the statistical analysis.

Differences were considered statistically significant if the corresponding *p*-value < 0.05.

## Results

A total of 4090 patients with a mean age of 67.45 ± 16.69 years (range: 5 to 99) were included in the present study. Among them, 62.2% were female with a mean age of 67.76 ± 16.13 years (range: 5 to 99) and 37.8% were male with a mean age of 66.32 ± 17.55 years (range: 8 to 97). Table 1 shows the mean and median values obtained in each eye for each parameter and the mean and median differences (left eye *vs.* right eye) for better visualization of the magnitudes.

**Table 1:** Descriptive and comparative analysis of those biometric parameters evaluated for the overall sample, showing for each parameter the mean and median values obtained in each eye, as well as the mean and median differences (left eye *vs.* right eye)

		AL (mm)	Rm (mm)	ACD (mm)	LT (mm)	CCT (mm)	W–W (mm)	CWC (mm)		
								CWD	x-CWC	y-CWC
OD	Average	23.91	7.70	3.18	4.51	553.64	11.94	0.35	0.30	−0.05
	STD	1.68	0.27	0.43	0.49	34.93	0.42	0.16	0.18	0.16
	Median	23.61	7.69	3.18	4.56	553.10	11.94	0.34	0.30	−0.05
	Max	34.41	9.61	4.69	6.13	696.57	13.56	1.64	1.32	1.28
	Min	19.16	6.62	1.71	3.05	432.65	10.17	0.01	−1.36	−1.50
OS	Average	23.86	7.69	3.18	4.51	553.88	11.96	0.32	0.26	−0.08
	STD	1.64	0.27	0.43	0.49	34.88	0.42	0.16	0.19	0.16
	Median	23.55	7.68	3.18	4.55	553.66	11.96	0.31	0.26	−0.07
	Max	35.38	8.98	4.62	6.33	692.20	13.51	2.03	2.01	1.42
	Min	19.29	6.80	1.84	3.06	426.90	10.14	0.01	−1.37	−1.46
OD vs OS comparison		< 0.001*	< 0.001*	0.012*	0.813	0.021*	< 0.001*	< 0.001*	< 0.001*	< 0.001*
OD–OS difference	Average	0.05	0.01	0.00	0.00	−0.24	−0.03	0.03	0.04	0.03
	STD	0.60	0.07	0.11	0.16	8.30	0.19	0.14	0.15	0.14
	Median	0.03	0.01	0.00	0.00	−0.28	−0.03	0.03	0.05	0.03
	Max	10.20	0.85	0.77	0.99	82.55	2.43	1.39	1.67	1.27
	Min	−4.05	−0.93	−1.10	−1.65	−109.87	−2.29	−1.51	−1.78	−1.45
ICC		0.935	0.962	0.965	0.948	0.972	0.896	0.612	0.656	0.597

*AL* axial length, *Rm* mean anterior curvature, *ACD* Anterior chamber depth, *LT* lens thickness, *CCT* central cornea thickness, *W–W* white-to-white or corneal diameter, *CWC* distance Chang–Waring chord, x-CWC and y-CWC: horizontal and vertical components for the Chang–Waring chord; *p < 0.05

ICC results are also included in Table 1, revealing an excellent correlation between the right eye and the left eye for almost all the biometric parameters under study (ICCs > 0.89) with the exception of the CWC, considering both its magnitude (CWC-D) and its vertical (y-CWC) and horizontal (x-CWC) components, which showed moderate correlation values (ICC = 0.612, ICC = 0.656 and ICC = 0.597) [11, 12].

When breaking the data down by gender, the results show a similar trend to that of the overall sample, as given in Table 2.

**Table 2:** The intraclass correlation coefficient (ICC) for female and male for the biometric parameters analyzed

	ICC	
	Female	Male
AL (mm)	0.924	0.948
Rm (D)	0.963	0.96
ACD (mm)	0.964	0.952
LT (mm)	0.948	0.947
CCT (mm)	0.974	0.968
WTW (mm)	0.898	0.884
CWC-D (mm)	0.626	0.588

*AL* axial length, *Rm* mean anterior curvature, *ACD* anterior chamber depth, *LT* lens thickness, *CCT* central cornea thickness, *WTW* white-to-white or corneal diameter, *CWC* distance Chang*–*Waring chord

**Fig. 1:**
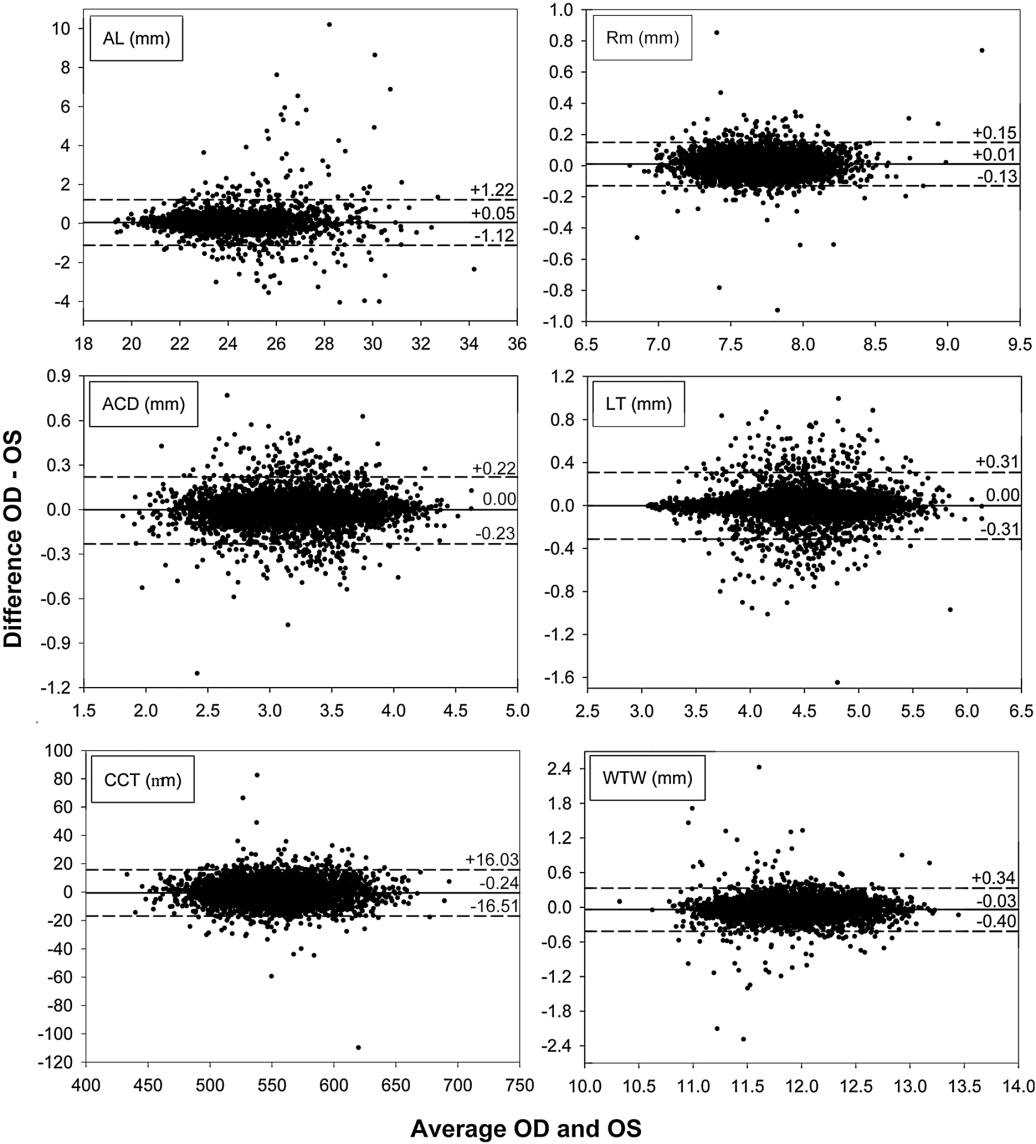
Bland–Altman graphs corresponding to each biometric parameter under study. The horizontal dashed lines represent the limits of agreement according to a 95% confidence interval around the mean value (horizontal solid line). *AL* axial length, *Rm* mean anterior curvature, *ACD* anterior chamber depth, *LT* lens thickness, *CCT* central cornea thickness, *WTW* white-to-white or corneal diameter

Figure 1 shows the Bland–Altman graphs corresponding to each biometric parameter under study. Mean differences OD–OS close to zero were obtained for all parameters. Limits of agreement for differences OD–OS for each variable are plotted in Fig. 1. The width of those limits of agreement around the mean value was 2.34 mm for AL, 0.28 mm for Rm, 0.45 mm for ACD, 0.62 mm for LT, 32.54 µm for CCT and 0.74 mm for WTW.

**Fig. 2:**
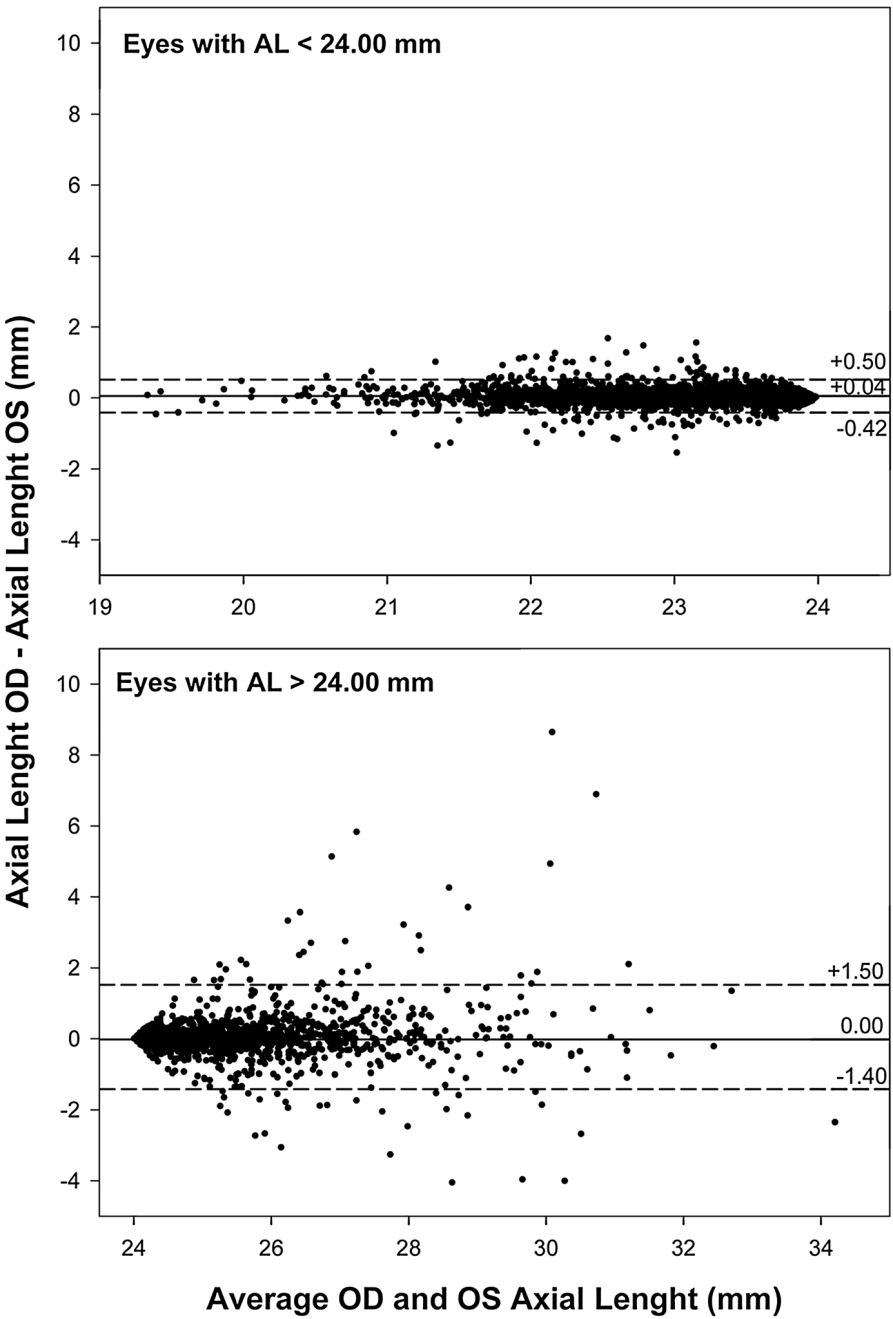
Bland–Altman graphs for the interocular difference in axial length (AL) for subjects with eyes shorter than 24 mm (top) and for eyes larger than 24 mm (bottom). The horizontal dashed lines represent the limits of agreement according to a 95% confidence interval around the mean value (horizontal solid line)

Bland–Altman plots revealed relevant differences only for AL, as shown in Fig. 1, with a width of more than 2 mm for the limits of agreement around the mean (OD–OS) value. The dispersion of differences in AL data shown in Fig. 1 appears to be concentrated in average ALs above 24 mm. If the sample for AL is broken into two groups (AL < 24 mm and AL ≥ 24 mm) and the Bland–Altman analysis is repeated, limits of agreement for the AL < 24 mm group narrow to 0.94 mm, whereas in the AL ≥ 24 mm group the width of the limits of agreement is 2.90 mm, as shown in Fig. 2. Axis scaling in Fig. 1 for AL is maintained in Fig. 2 for better visualization of the differences in the width of the limits of agreement.

Table 3 shows the results of the Passing–Bablok regression. The linearity of the OD-*vs-*OS relationship can be correctly assumed for all parameters (Cusum test: *p* > 0.05 in all cases). Confidence intervals were obtained for the slope containing the unit value, while the confidence interval for “intercept” contains the zero value for all parameters except for WTW and CWC.

**Table 3:** Results of the Passing–Bablok regression comparing values obtained between right and left eye for every parameter

	Regression equation	95% CI for intercept		95% CI for slope		CTL
AL	OD = 0.0990 + 0.9971 OS	−0.0370	0.2340	0.9914	1.0029	*p* = 0.99
Rm	OD = 0.0457 + 0.9951 OS	−0.0133	0.1040	0.9875	1.0028	*p* = 0.32
ACD	OD = −0.0168 + 1.0044 OS	−0.0370	0.0033	0.9981	1.0107	*p* = 0.42
LT	OD = −0.0111 + 1.0027 OS	−0.0351	0.0123	0.9973	1.0081	*p* = 0.52
CCT	OD = −1.3743 + 1.0020 OS	−5.2811	2.4463	0.9950	1.0090	*p* = 0.62
WTW	OD = −0.1561 + 1.0108 OS	−0.2913	−0.0233	0.9997	1.0220	*p* = 0.25
x-CWC	OD = 0.0452 + 0.9994 OS	0.0392	0.0508	0.9755	1.0238	*p* = 0.80
y-CWC	OD = 0.0313 + 1.0020 OS	0.0293	0.0331	0.9731	1.0316	*p* = 0.62

*AL* axial length, *Rm* mean anterior curvature, *ACD* anterior chamber depth, *LT* lens thickness, *CCT* central cornea thickness, *W–W* white-to-white or corneal diameter, *x-CWC and y-CWC* horizontal and vertical components for the Chang–Waring chord, *CI* confidence interval, *CTL* Cusum test for linearity (linearity if *p* > 0.05)

**Fig. 3:**
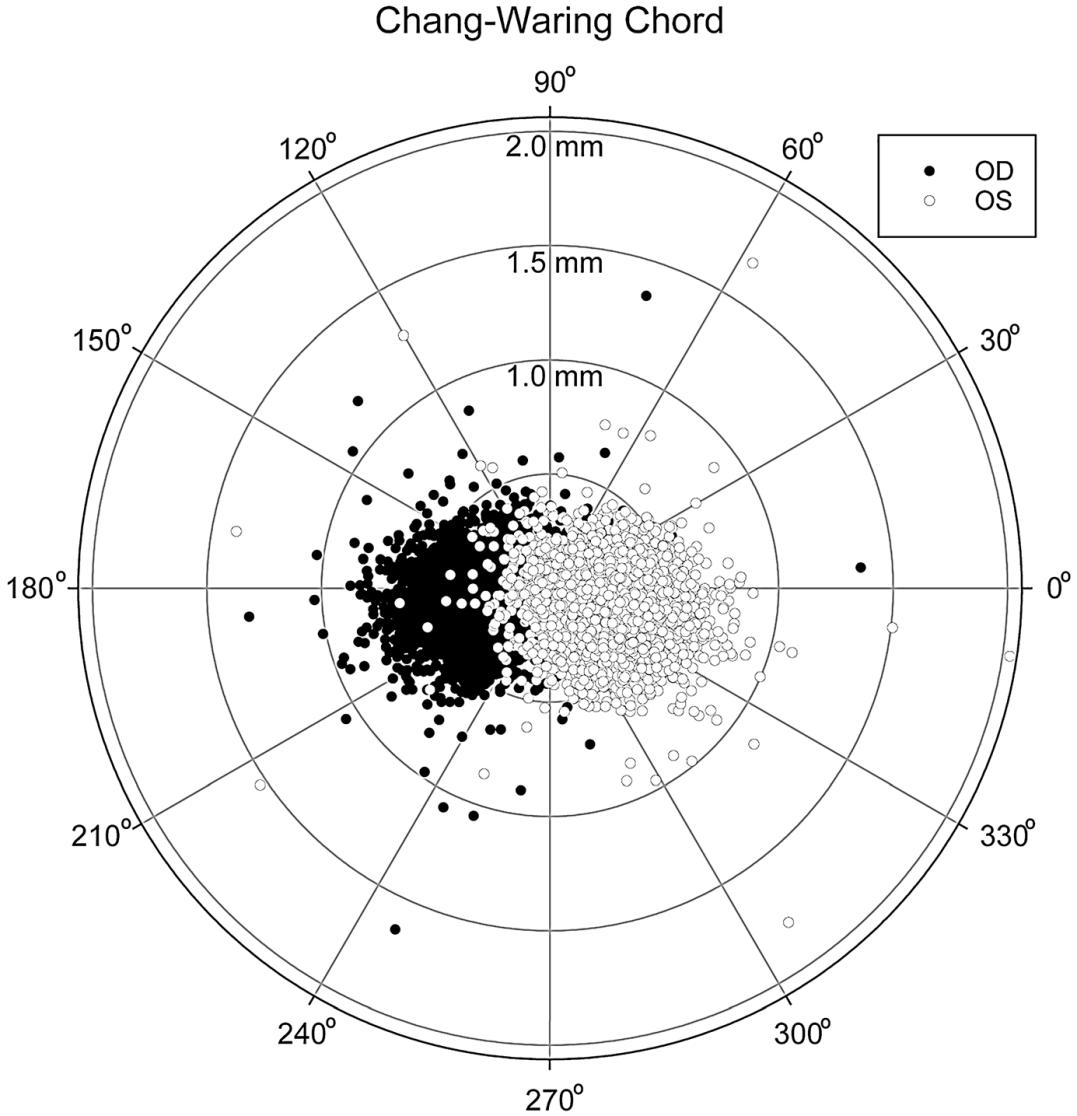
Polar coordinate diagram showing Chang–Waring chord (CWC) values for both eyes (black figures for OD and white figures for OS). *OD* right eyes, *OS* left eyes

**Fig. 4:**
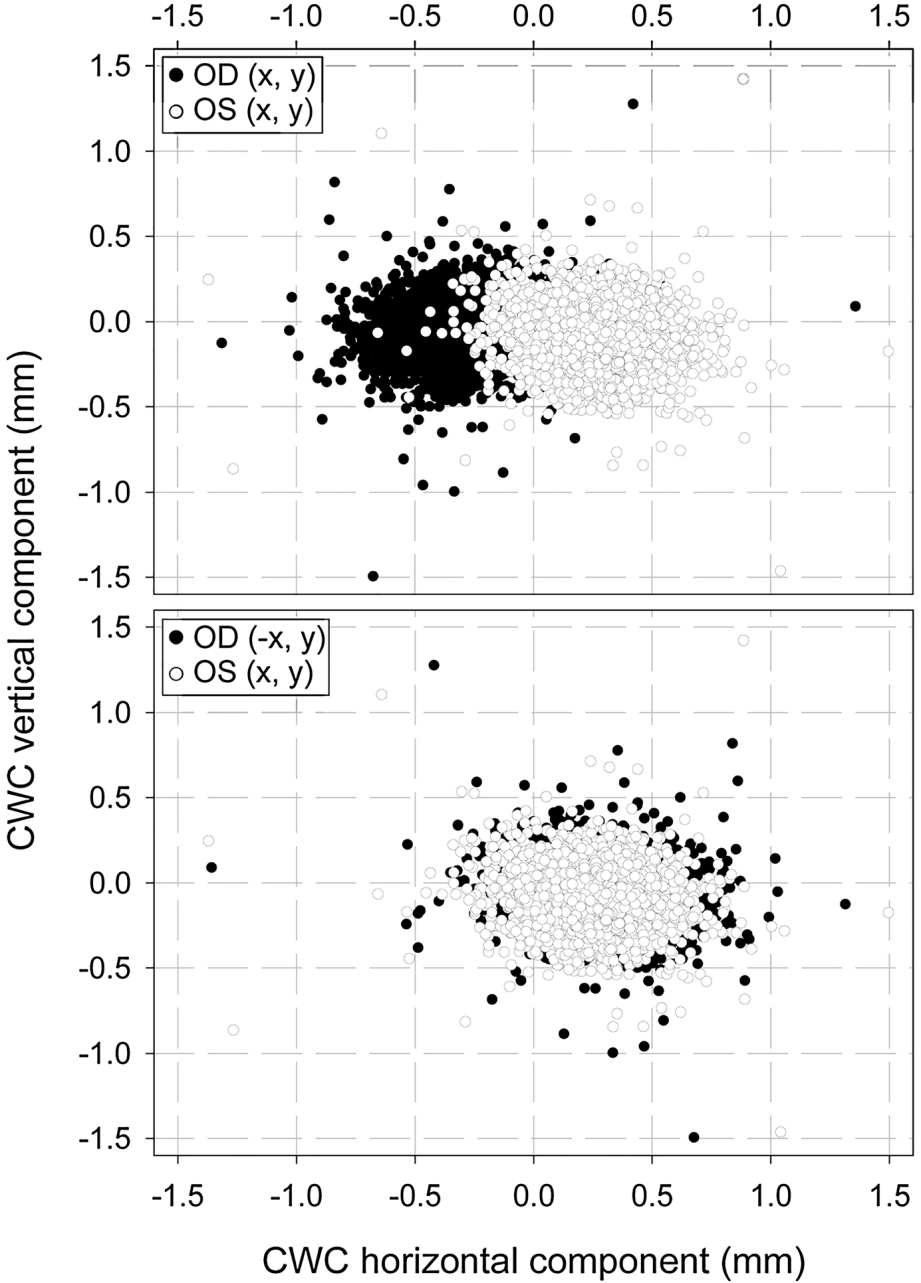
Cartesian coordinate diagram showing CWC components for both eyes (black figures for OD and white figures for OS). For the right eyes (OD), the sign of the x-coordinate has been reversed to remove the effect of specularity (bottom)

The Chang–Waring chord (CWC) analysis was performed separately from the rest of the parameters due to its vector nature. Values obtained for both eyes are shown in Fig. 3, which is a polar-coordinate plot, where the specular symmetry between left and right eyes can be seen. In order to statistically evaluate the potential differences between left and right eyes, CWC values (distance and angle) were converted into their corresponding *x* and *y* Cartesian components. Then, aiming to remove the effect of specular symmetry, we reversed the sign of the *x*-coordinate value for all the right eyes, thus obtaining the results shown in Fig. 4. Despite the nearly perfect symmetry that Figs. 3 and 4 seem to show, the statistical analysis reveals OD-*vs-*OS differences for the CWC's *x* and *y* components (*p* < 0.001 for both coordinates).

## Discussion

The results of the present study confirm that our 4090 patient population show excellent ocular symmetry in terms of Rm, ACD, LT, CCT and W–W, as well as moderate ocular symmetry for CWC-D, as assessed by means of the swept-source optical biometry system IOLMaster 700 (Carl Zeiss Meditec AG, Jena, Germany). AL showed good agreement for eyes shorter than 24 mm and only moderate agreement for larger eyes.

There is some controversy regarding the impact of the interocular (i.e., left-*vs*-right) differences shown by certain parameters—such as AL and corneal power—upon postoperative refractive outcomes [13, 14]. Different studies have tackled bilateral symmetry by using the IOLMaster 500 [14–18], Pentacam [15, 19–23] and Orbscan II biometers [3, 24] (among others), but we have not found any studies in the literature where bilateral symmetry had been assessed by means of the swept-source optical biometric system that the IOLMaster 700 biometer includes.

All biometric parameters analyzed in our study, except LT, showed interocular differences with corresponding *p*-value < 0.05. However, we should not focus solely on statistical significance, but we must also assess whether or not those differences are clinically relevant. In this regard, it is important to consider also the ICC analysis, the Passing–Bablok regression and the Bland–Altman analysis, all of which help to draw more robust conclusions regarding the bilateral symmetry of the biometric parameters under study. It is also important to consider the repeatability and the reproducibility of the measurements [9].

### Axial length

Axial length’s bilateral symmetry was analyzed by Li et al., in 2014, in 397 subjects, using an A-scan ultrasound device (Compuscan UAB 1000, Storz Inc., St. Louis, MO). The study revealed an excellent correlation between both eyes (*r* = 0.95) [25]. Regarding studies using the IOLMaster 500 biometer, they have shown similar results to those of the present study, especially for eyes shorter than 24 mm: For instance, in a sample of 50 healthy subjects, mean interocular AL difference was as low as 0.01 ± 0.32 mm, showing a strong interocular AL correlation (*r* = 0.96) [26]. Hong et al. [17] also found bilateral symmetry in terms of AL when assessing a sample of 260 healthy subjects (22.38 ± 4.1 years). Mean interocular difference was 0.003 ± 0.217 mm (*p* = 0.839). Another multicenter study encompassing 14,016 subjects also revealed interocular symmetry for AL, with a mean difference of 0.06 ± 0.40 mm [27]. Compared to the above-mentioned research studies, ours yielded a high interocular correlation for AL (ICC = 0.935), while the Bland–Altman analysis (Fig. 1) showed +1.22 mm and −1.12 mm as upper and lower 95% limits around mean difference (0.05 ± 0.60 mm). The Passing–Bablok regression analysis allows us to conclude the equivalence of the left-eye and right-eye AL values, with a better agreement for eyes shorter than 24 mm, as shown in Fig. 2.

However, there are also some authors who did report bilateral asymmetry for AL, for instance, Mahroo et al. [16], who assessed a sample of 4571 subjects (75.9 ± 9.4 years) with bilateral cataract and concluded that the right eyes were, on average, 0.05 mm longer than the left ones (*p* < 0.001). Although this 0.05-mm difference has a significantly associated *p*-value, it cannot be considered clinically significant unless a proper correlation analysis is subsequently performed. For eyes having AL > 24.8 mm, more relevant interocular differences have been observed. Kansal et al. [14] reported a mean interocular difference of 0.36 ± 0.41 mm for a sample of 389 myopic subjects, while the results for a sample of 202 subjects with high bilateral myopia showed bilateral symmetry, with mean AL for left and right eyes of 29.43 ± 2.40 mm and 29.31 ± 2.39 mm, respectively (*p* = 0.587) [15].

Regarding the results obtained by Kansal et al. [14] for eyes with AL > 24.8 mm, our results show a trend to more interocular differences in AL for larger eyes. As shown in Fig. 1, the differences in AL for eyes shorter than 22 mm are insignificant, whereas larger differences appear for eyes larger than 24 mm (especially between 26 and 31 mm).

In summary, no overall interocular differences have emerged from our study for axial length in eyes shorter than 24 mm, and moderate differences have been found for eyes larger than 24 mm. Nonetheless, according to Knox Cartwright et al.’s [27] conclusions, when left-to-right-eye AL difference exceeds 0.70 mm for a given subject, it is recommended to verify biometric data to minimize the likelihood of post-surgical refractive errors.

### Mean anterior curvature (Rm)

The cornea provides approximately two-thirds of the eye’s refractive power [28]. The interocular differences for the anterior corneal power have been studied by other authors and the vast majority of them observed symmetry for this biometric parameter using the Pentacam system. Li et al. [25] analyzed ocular symmetry in terms of anterior corneal curvature (D), finding a mean interocular difference of 0.00 ± 0.24 D (*p* = 0.939) and a high correlation (*r* = 0.99). Dienes et al. [21] also studied ocular symmetry with the Pentacam system in 65 healthy subjects (mean age: 39.95 ± 15.44 years) and reported mean differences of 0.37 ± 0.39 mm and 0.43 ± 0.44 mm for the flat and steep meridians, respectively. A study by Naderan et al. [29] on 306 healthy eyes also found no interocular differences for mean anterior corneal power (43.0 ± 1.1 D and 43.2 ± 1.2 D for right and left eyes, respectively (*p* = 0.325)). Another multicenter study with interocular analysis of 14,016 subjects reported interocular symmetry with differences of −0.07 ± 0.49 mm [27].

Our results are in good agreement with those reported by the above-mentioned authors; moreover, considering the IOLMaster 700’s repeatability and reproducibility features for keratometric radius (0.02 ± 0.06 mm and 0.02 ± 0.07 mm, respectively) [9], we have found bilateral symmetry for Rm despite the fact that *p*-value < 0.05. The Passing–Bablok analysis places the values 1 and 0 within the 95% confidence intervals for “slope” and “intercept,” respectively, which means that equivalence can be assumed based on the Rm values obtained for left and right eyes.

### Anterior chamber depth (ACD)

ACD is another important IOL power calculation biometric parameter. Olsen et al. [30] reported refractive error rates as high as 42, 36 and 22% resulting from mistakes when measuring ACD, AL and corneal power, respectively.

Our results suggest there is bilateral symmetry for ACD, despite the *p*-value being <0.05. Considering that the IOLMaster 700’s repeatability and reproducibility features for ACD are 0.01 ± 0.02 mm and 0.01 ± 0.03 mm, respectively [9], minor changes to those values cannot be considered to be relevant. The Passing–Bablok analysis places the values 1 and 0 within the 95% confidence intervals for “slope” and “intercept,” respectively, which means that equivalence can be assumed based on the ACD values obtained for left and right eyes.

Other authors have also studied ACD’s bilateral symmetry using different measuring devices. Li et al. [25] used an ultrasonic biometer (an A-scan ultrasound device [Compuscan UAB 1000, Storz Inc., St. Louis, MO]) in 397 subjects, and they found a mean interocular difference of −0.01 ± 0.15 mm with a high Pearson’s correlation coefficient (*r* = 0.86). Palamar et al. [22] obtained a similar mean difference (−0.03 ± 0.07 mm) using a different A-scan biometer model (Sonogage Eye Scan, Cleveland, OH, USA) in a population comprising 42 hyperopic subjects showing anisometropia (1 D mean spherical equivalent difference between eyes).

### Crystalline lens thickness (LT)

This biometric parameter has been much less studied than AL, ACD and Rm. In the above-mentioned study on hyperopic eyes having anisometropia (1 D mean spherical equivalent difference between eyes), mean interocular difference for LT was −0.09 ± 0.40 mm, their population sample being younger and smaller than our study’s [22]. Moreover, these authors found no differences between eyes having different AL. (For their sample, mean interocular difference for AL was −0.95 ± 0.50 mm.) Similarly, there are other authors that found no interocular differences for LT either (−0.01 ± 0.45 mm), although in this case Pearson’s correlation analysis revealed a weak correlation (*r* = 0.37) [25]. On the other hand, the results obtained in the present study—namely mean difference (0.00 ± 0.16 mm), ICC (0.948) and Bland–Altman graphs—underpin interocular symmetry for lens thickness. The Passing–Bablok analysis places the values 1 and 0 within the 95% confidence intervals for “slope” and “intercept,” respectively, which means that equivalence can be assumed based on the LT values obtained for right and left eyes.

### Central corneal thickness (CCT)

Bilateral symmetry for CCT was confirmed in our study. Some studies in the literature have analyzed the interocular CCT difference using the Orbscan system, but most have relied on the Pentacam platform, which has a measurement variability of 0.51 μm, as reported by some authors [31]. Thus, Dienes et al. [21] and Henriquez et al. [23] studied ocular symmetry both in eyes with keratoconus and in healthy eyes. In Henriquez et al.’s [23] healthy-eye sample (53 subjects; mean age 28.4 ± 5.3 years), mean interocular difference was 10.28 ± 7.89 μm, while in Dienes et al.’s [21] (65 subjects; mean age 39.95 ± 15.44 years), mean interocular difference was 5.59 ± 4.90 μm, showing an excellent correlation between both eyes (*r* = 0.98). The study by Li et al. [19] produced the same correlation value (*r* = 0.98) although with a smaller interocular difference (0.51 ± 6.79 μm). Falavarjani et al. [20], using a larger sample (275 subjects; mean age 29.1 ± 7.73 years), also obtained an excellent correlation (*r* = 0.90) with a mean interocular difference of 8.42 μm.

Durr et al. [24] studied interocular differences in 3835 subjects (a sample size similar to ours) with the Orbscan system, obtaining a mean difference for CCT of 0.28 μm, with ICC = 0.984 [24]. Other authors who also relied on the same measuring platform found a mean interocular difference of 8 ± 7 μm, with a high correlation (*r* = 0.95) [3]. In this context, our results resemble those obtained by Durr et al. [24] and Li et al. [25]: mean difference of 0.24 μm and ICC = 0.972.

### Chang–Waring chord distance (CWC-D) and Chang–Waring chord angle (CWC-A)

No studies have been found in the literature analyzing interocular differences for these two parameters (CWC-D and CWC-A). Our results have shown a mean interocular difference of 0.03 ± 0.14 mm (*p* < 0.05) for CWC-D, with moderate interocular correlation. When the CWD vector is broken down into its horizontal and vertical components and these values are plotted in a graph, a quasi-perfect specular symmetry is observed (Figs. 3 and 4), although the statistical analysis did reveal differences between both eyes.

### White-to-white distance (WTW)

Measuring the horizontal corneal diameter—the so-called white-to-white distance (WTW)—is required for cataract and refractive surgery and also for the diagnosis and characterization of certain corneal pathologies. Interocular differences for WTW have been studied using the Orbscan II system in a sample of 1001 subjects aged 18 to 45 years. Authors found no interocular differences (*p* = 0.26), reporting mean WTW of 11.66 ± 0.37 mm and 11.66 ± 0.31 mm for right and left eyes, respectively [32]. In a sample of 231 myopic subjects, no interocular differences were found either [2]. Our results are in line with the previous studies and confirm the bilateral symmetry for the WTW parameter. Despite the statistically significant differences, we can conclude that the difference of 0.03 mm between left and right eyes obtained in this study is not clinically relevant, taking into account that repeatability and reproducibility for this parameter amounts to 0.10 ± 0.29 and 0.13 ± 0.35 mm, respectively [9].

## Conclusions

Analysis of our results in 4090 subjects leads us to conclude that there are no clinically significant interocular differences for the biometric parameters under study—AL, Rm, CD, LT, CCT, WTW and CWC-D—although for all of them, except the LT, statistically significant differences did arise. Regarding CWD, the results have revealed clinically significant differences although it must be said that they are related to enantiomorphism. In the case of AL, moderate differences can be expected in eyes larger than 24 mm.

In the event, for instance, of a cataract campaign in developing countries, if keratometry in one eye cannot be obtained, the other eye’s value can be used. However, this strategy procedure must be carefully performed regarding axial length, especially in larger eyes.

The behavior for the overall sample was similar to the one observed after breaking the data down by gender.

## Declarations

### Data availability

Data can be requested from the Miranza R&D Department (info.ioa@miranza.es).

## References

[1] Wang L, Dai E, Koch DD, Nathoo A (2003). Optical aberrations of the human anterior cornea. J Cataract Refract Surg.

[2] Zha Y, Feng W, Han X, Cai J (2013). Evaluation of myopic corneal diameter with the Orbscan II topography system. Graefe's Arch Clin Exp Ophthalmol.

[3] Myrowitz EH, Kouzis AC, O'Brien TP (2005). High interocular corneal symmetry in average simulated keratometry, central corneal thickness, and posterior elevation. Optometry Vis Sci Official Public Am Acad Optometry.

[4] Choi Y, Eom Y, Song JS, Kim HM (2017). Influence of corneal power on intraocular lens power of the second eye in the SRK/T formula in bilateral cataract surgery. BMC Ophthalmol.

[5] Jabbour J, Irwig L, Macaskill P, Hennessy MP (2006). Intraocular lens power in bilateral cataract surgery: whether adjusting for error of predicted refraction in the first eye improves prediction in the second eye. J Cataract Refract Surg.

[6] Covert DJ, Henry CR, Koenig SB (2010). Intraocular lens power selection in the second eye of patients undergoing bilateral, sequential cataract extraction. Ophthalmology.

[7] Aristodemou P, Knox Cartwright NE, Sparrow JM, Johnston RL (2011). First eye prediction error improves second eye refractive outcome results in 2129 patients after bilateral sequential cataract surgery. Ophthalmology.

[8] Chiang PK, Hersh PS (1999). Comparing predictability between eyes after bilateral laser in situ keratomileusis: a theoretical analysis of simultaneous versus sequential procedures. Ophthalmology.

[9] Bullimore MA, Slade S, Yoo P, Otani T (2019). An evaluation of the IOLMaster 700. Eye Contact Lens.

[10] Chang DH, Waring GOt (2014). The subject-fixated coaxially sighted corneal light reflex: a clinical marker for centration of refractive treatments and devices. Am J Ophthalmol.

[11] Bland JM, Altman DG (1996). Measurement error and correlation coefficients. BMJ.

[12] Ludbrook J (2002). Statistical techniques for comparing measurers and methods of measurement: a critical review. Clin Exp Pharmacol Physiol.

[13] Rajan MS, Bunce C, Tuft S (2008). Interocular axial length difference and age-related cataract. J Cataract Refract Surg.

[14] Kansal V, Schlenker M, Ahmed IIK (2018). Interocular axial length and corneal power differences as predictors of postoperative refractive outcomes after cataract surgery. Ophthalmology.

[15] Zhu X, He W, Du Y, Zhang K, Lu Y (2019). Interocular symmetry of fixation, optic disc, and corneal astigmatism in bilateral high myopia: the shanghai high myopia study. Transl Vis Sci Technol.

[16] Mahroo OA, Williams C, Hysi PG, Williams KM, Kailani O, Thompson J, Cumberland PM, Guggenheim JA, Rahi JS, Harrad RA, Hammond CJ (2015). Interocular asymmetries in axial length and refractive error in 4 cohorts. Ophthalmology.

[17] Hong SW, Lee SB, Jee DH, Ahn MD (2015). Interocular retinal nerve fiber layer thickness difference in normal adults. PLoS ONE.

[18] Parssinen O, Kauppinen M, Halekoh U, Kaprio J, Rantanen T (2019). Heredity of interocular similarities in components of refraction: a population-based twin study among 66- to 79-year-old female twins. Acta Ophthalmol.

[19] Senthilkumari S, Talwar B, Dharmalingam K, Ravindran RD, Jayanthi R, Sundaresan P, Saravanan C, Young IS, Dangour AD, Fletcher AE (2014). Polymorphisms in sodium-dependent vitamin C transporter genes and plasma, aqueous humor and lens nucleus ascorbate concentrations in an ascorbate depleted setting. Exp Eye Res.

[20] Falavarjani KG, Modarres M, Joshaghani M, Azadi P, Afshar AE, Hodjat P (2010). Interocular differences of the Pentacam measurements in normal subjects. Clin Exp Optom.

[21] Dienes L, Kranitz K, Juhasz E, Gyenes A, Takacs A, Mihaltz K, Nagy ZZ, Kovacs I (2014). Evaluation of intereye corneal asymmetry in patients with keratoconus. A Scheimpflug imaging study. PLoS ONE.

[22] Palamar M, Degirmenci C, Biler ED, Egrilmez S, Uretmen O, Yagci A (2016). Evaluation of the anatomic and refractive differences in hyperopic anisometropia. Int Ophthalmol.

[23] Henriquez MA, Izquierdo L, Mannis MJ (2013). Intereye asymmetry detected by Scheimpflug imaging in subjects with normal corneas and keratoconus. Cornea.

[24] Durr GM, Auvinet E, Ong J, Meunier J, Brunette I (2015). Corneal shape, volume, and interocular symmetry: parameters to optimize the design of biosynthetic corneal substitutes. Invest Ophthalmol Vis Sci.

[25] Li Y, Bao FJ (2014). Interocular symmetry analysis of bilateral eyes. J Med Eng Technol.

[26] Chen FK, Yeoh J, Rahman W, Patel PJ, Tufail A, Da Cruz L (2012). Topographic variation and interocular symmetry of macular choroidal thickness using enhanced depth imaging optical coherence tomography. Invest Ophthalmol Vis Sci.

[27] Knox Cartwright NE, Johnston RL, Jaycock PD, Tole DM, Sparrow JM (2010). The Cataract National Dataset electronic multicentre audit of 55567 operations: when should IOLMaster biometric measurements be rechecked?. Eye.

[28] DelMonte DW, Kim T (2011). Anatomy and physiology of the cornea. J Cataract Refract Surg.

[29] Naderan M, Rajabi MT, Zarrinbakhsh P (2017). Intereye asymmetry in bilateral keratoconus, keratoconus suspect and normal eyes and its relationship with disease severity. Br J Ophthalmol.

[30] Olsen T (2007). Calculation of intraocular lens power: a review. Acta Ophthalmol Scand.

[31] Chen D, Lam AK (2007). Intrasession and intersession repeatability of the Pentacam system on posterior corneal assessment in the normal human eye. J Cataract Refract Surg.

[32] Gharaee H, Abrishami M, Shafiee M, Ehsaei A (2014). White-to-white corneal diameter: normal values in healthy Iranian population obtained with the Orbscan II. Int J Ophthalmol.

